# Public Health Aspects in the Prevention and Control of Vitamin Deficiencies

**DOI:** 10.1093/cdn/nzz075

**Published:** 2019-06-21

**Authors:** Ian Darnton-Hill

**Affiliations:** 1 The Boden Institute of Obesity, Nutrition, Exercise & Eating Disorders, Charles Perkins Centre, Faculty of Medicine, University of Sydney, New South Wales 2006, Australia; 2 The Friedman School of Nutrition Science and Policy, Tufts University, Boston, MA 021111, USA

**Keywords:** vitamins, vitamin deficiencies, prevention and control, maternal, infant and young child nutrition

## Abstract

Vitamin deficiencies remain major etiological factors in the global burden of disease, especially in low- and middle-income countries. The purpose of this state-of-the-art review was to update current information on deficiencies of vitamins and public health approaches to addressing them. Some stages of life present a higher risk of deficiency than others: risks are higher in pregnant women, children (from conception to young childhood), adolescents, the elderly, and all of the over 800 million people globally who are undernourished. At risk are approximately 125 million preschool children with vitamin A deficiency, as well as sub-populations at risk of deficiencies of folate, thiamin, vitamin B12, niacin, riboflavin, other B vitamins. and vitamin D. Addressing micronutrient deficiencies requires identifying those at risk and then working to prevent and manage that risk. Public health approaches include improved, diversified diets; supplementation; fortification and biofortification; and other supportive public health measures. Historically, as with pellagra and beriberi and, in the last 3 decades, with vitamin A and folic acid, there has been encouraging progress, but much remains to be done.

## Introduction

Globally, the WHO is frequently quoted as estimating that almost 2 billion people are at risk of micronutrient deficiencies (vitamins and minerals/trace elements) (1). Amongst these are approximately 125 million preschool children with vitamin A deficiency, as well as sub-populations at risk of deficiencies of folate, thiamin, vitamin B12, niacin, other B vitamins, and vitamin D (1, 2). By their very definition, vitamins are essential for optimizing health and, indeed, for life. Some stages of life present a higher risk of deficiency than others: risks are higher in pregnant women, children (from conception to young childhood), adolescents, and the elderly. The latest estimate by 5 UN agencies is that 821 million people globally are undernourished, which puts them at risk of vitamin and other micro- and macro-nutrient deficiencies (3).

### Background

This brief state-of-the-art review of much of the recent literature looks at vitamin deficiencies throughout the life cycle, including vitamin deficiencies in pregnancy and the first 1000 days after birth, particularly in low- and middle-income countries (LMICs). Public health approaches to vitamin deficiency prevention and control have developed and expanded over the last 4 decades (August 8-10 in Sydney, Australia). However, with an increasing number of programs using different approaches, and also increased availability due to commercial fortification, there is also at least a theoretical possibility of overlap, with excess intakes of some vitamins (4, 5). The US National Institutes of Health, through the Biomarkers for Nutrition in Development (BOND) Initiative (6), have convened up-to-date expert reviews of folate (7), vitamin A (8), and vitamin B12 (9) (and 3 minerals), showing, amongst other things, the still pressing need for more knowledge on biomarkers, cutoffs, and the need for more data on the prevalences of deficiencies, especially in LMIC. More recently, the New York Academy of Sciences reviewed thiamin and vitamin D (10–12), and before that the European Community established up-to-date reviews and recommendations through activity from European Micronutrient Recommendations Aligned (13).

Basically, vitamins are a group of organic compounds that are essential for normal growth and optimal nutrition and are required in small quantities in the diet because they cannot be synthesized by the body. As is well-known, the word “vitamine” was coined in 1911 by the Warsaw-born biochemist Casimir Funk (1884–1967). At the Lister Institute in London, Funk isolated a substance that prevented nerve inflammation (neuritis) in chickens raised on a diet deficient in that substance, which he named “vitamine” because he believed it was necessary to life and it was a chemical amine. The “e” at the end was later removed when it was recognized that vitamins need not be amines (14); this was also true of thiamine (also thought to be an amine), now more correctly identified as thiamin (15). The letters (A, B, C, and so on) were assigned to the vitamins in the order of their discovery. The single exception was vitamin K, which was assigned its “K” from “Koagulation” by the Danish researcher Henrik Dam. Many of the physiologic functions of vitamins are known, but much is not (2, 6–9). Most of the vitamins are recognized as being of public health significance (vitamin A, or retinol, and the pro-vitamin A, beta carotene; B vitamins, such as folate, B12, and others, including vitamin B1, or thiamin; vitamin B2, or riboflavin; vitamin B3, or niacin; vitamin B6, or pyridoxine; and, less so, vitamin C, or ascorbic acid; vitamin E; and vitamin K), all of which have co-enzyme or co-factor actions in biochemical and physiological functions for the body's functioning, growth, and maintenance. Many also have more specific actions as well; for example, vitamin A has a role in preventing blindness, folate and vitamin B12 are important factors in nucleic acid synthesis, vitamin C has importance in the synthesis of collagen, vitamin K is an essential factor in the formation of blood clotting factors, and so on. Vitamin D is actually a steroid vitamin that promotes the absorption and metabolism of calcium and phosphorus (and in some settings is also sourced largely from exposure to sunlight). Folate, niacin, riboflavin, and vitamins B6 and B12, as well as zinc, are all involved in 1-carbon metabolism and, thus, are important in early gestation and early cell proliferation, growth, and protein synthesis (16, 17).

### Scope of review

This state-of-the art review looked at the impacts of vitamin deficiencies; their prevalences in terms of population risks; methods used in the prevention and control of such deficiencies; political, social, and other factors; current coverage and progress; some emerging issues; and some conclusions. As noted, the emphasis is on LMICs and programmatic aspects. The review used a wide-ranging search for references in both the formal literature and the grey literature (including by Non-Government Organisations), mainly by “snowballing” (forward citation searching): that is, identifying relevant references of key articles, following them up, and then repeating the process with each article used. This was underpinned by a formal literature search of articles and reports over the last 15 years (2000–2015), in English only, through the University of Sydney Fisher Library, with an emphasis on micronutrient programs and fortification. The keywords used were: “Fortified food^*^,” “Enriched food^*^,” “Supplemented food^*^,” and, for medical subject headings in Medline (via OvidSP), “Food, fortified//ae- adverse effects.” The complementary keywords also used were: “Government program^*^,” “Government sponsored program^*^,” “Nutritional policy,” “Government health promotion,” “Food fortification program^*^,” “Policymaker^*^,” “Health polic^*^,” “Mandatory program^*^,” and, under medical subject headings, “Health promotion/og,” “Nutrition policy/,” “Health policy/,” “Mandatory programs/,” “Policy making,” and “Legislation, Food/.” Over the prior 4 years, these searches were updated through weekly reviews of relevant journals, including the *American Journal of Clinical Nutrition*, *Bulletin of the WHO*, *Asian Pacific Journal of Clinical Nutrition*, *Journal of Nutrition*, *PLOS One*, and so on, and with periodic updates of the original search. Clarifications and unpublished data from persons involved in global micronutrient program activities were also sought. Finally, only those articles with emphases on vitamins (as opposed to micronutrients in general) were selected, as well as those where information on vitamins was thought to be relevant and was extracted.

## Impacts of Vitamin Deficiencies

The health of women, as well as their reproductive outcomes, are particularly affected by vitamin deficiencies, even more than had generally been considered (18–20). Similarly, the social impact of the health consequences of vitamin deficiencies is also disproportionately borne by women (21, 22), adolescents, and children (7, 22), according to socio-economic strata (23). As infants and young children grow rapidly, their immune systems, incidences of infectious diseases, intellectual development, physical development, and growth can all be affected by vitamin deficiencies (2). The elderly, as a sub-population, are often at risk (7, 24). Increasingly, it is clear that also there are interactions between vitamin deficiencies and chronic diseases, including deficiencies in obese individuals and those with lower bioavailability: these are not always identified clinically (25). On a larger scale, vitamin deficiencies contribute to the global burden of disease, economic costs, and constrained national development (26), although the overall impact of vitamins alone is not known and likely to be small. Nevertheless, it has been estimated that the burden of micronutrients in general, using the 2 billion figure, leads to the risk of suboptimal development in 40–60% of children in the 6- to 24-month age group who are growing up in LMICs (27).

A decline in dietary quality, leading to deficiencies in vitamins (and other micronutrients), is an early response to crises that affect food supplies and generate household food insecurities, and so, evidence of vitamin deficiencies has been used as an early indicator in such crises (28, 29). Although historical and well-recognized impacts of vitamin deficiencies are used in the development of programs, emerging examples of underestimated, subtle, and uncertain impacts of some vitamin deficiencies (e.g., vitamin B12, vitamin D, and so on) are increasingly being recognized (6, 30). Vitamin D is essential for normal metabolism, and some recent evidence suggests that inadequate concentrations of vitamin D may be associated with weight gain, obesity, the metabolic syndrome, and even cancer (31), particularly as low-serum vitamin D in US adolescents has been associated with hypertension, hyperglycaemia, and the metabolic syndrome, independent of adiposity (30). The recent publication of the results of the large, multicenter Vitamin D and Lifestyle (VITAL) study of supplementation with vitamin D did not, however, result in lower incidences of invasive cancer or cardiovascular events than a placebo (32). A high frequency of vitamin D deficiency in currently pregnant Japanese women has been associated with ultraviolet radiation avoidance and a diet low in vitamin D (33). Vitamins A, D, E, and B12 and folate all contribute toward protection against oxidative stress, enhance immune cell proliferation, regulate epithelial integrity, and improve antigen action (34, 35).

### Prevalence of population risk and scope of the problem

Even within the last millennium, micronutrient deficiencies were also common in industrializing countries of European and North American countries, at least in the more deprived sub-populations (2, 36). Vitamin A deficiency was widespread in Europe, and notably in Denmark, where it contributed to deaths in young children. Rickets was common in children who lived in the industrial cities of North America and Europe from the 17th until the early 20th centuries, when over 85% of the children living in these areas had rickets, primarily due to a lack of sunshine and insufficient production of vitamin D in the skin (36). Almost as soon as vitamin D was synthesized in the 1930s, it was used to fortify milk in Europe and North America, leading to the eradication of rickets as a major health problem in children. In the early 20th century, pellagra was common in the maize-eating populations of the southeast United States. At the peak of the epidemic (1928**–**30), 7000 individuals died each year from pellagra due to niacin deficiency (2, 36).

The common estimate of those suffering from micronutrient malnutrition (deficiencies of vitamins and trace elements) is around 2000 million people (1). Single micronutrient deficiencies rarely happen in isolation; more often, multiple deficiencies are occurring simultaneously, especially in women in LMICs globally (18–20). More specifically, current estimates are that vitamin A deficiency is present in ∼190 million (1 in 3) preschool children (8); folate deficiency is present in 15–49% pre-fortification (7); and vitamin D is a global problem impacting up to 1 billion people (30); with less clear or localized estimates for vitamin B12, thiamin, other B vitamins, and vitamin E (2). The 2 billion figure includes the iodine deficiency disorders, with an estimate of approximately 1.88 billion inadequate intakes, zinc deficiencies, and anemias caused by micronutrient deficiencies, predominantly of iron, but also of folate, vitamin B12, other B vitamins (2), vitamin A (37), and vitamin E (38), depending on the context (39).

Other, less-common vitamin deficiencies have still been reported but have usually been present in specific circumstances, such as in refugee camps and in those with restricted diets: there were reports of an outbreak of beriberi among African Union troops in Mogadishu, Somalia, who were living on restricted rations, which was quickly responsive to thiamin supplementation (40). Deficiencies of some of the other B vitamins, while not uncommon in LMICs, are rarely life-threatening (except thiamin deficiency in some circumstances) (2, 10). Nevertheless, because of their role in serious deficiency diseases such as pellagra (a deficiency of niacin) and anemia (riboflavin and other B vitamins), as well as superficial skin and mouth lesions (2), these B-vitamins are added to cereal fortifications and supplementary foods. Riboflavin deficiency is known to be endemic in many LMICs (41). There is also evidence that riboflavin’s status is generally low in the UK population and other affluent countries, and particularly in the elderly and in younger women (42).

Vitamin B12 deficiency is usually underestimated, but has been described as “a major public health problem worldwide” (43) and has been reported to be as high as 40% in Latin America, 70% in Africa, and 70–80% in South Asia (35). The authors of a recent review suggested that vitamin B12 deficiency increases the risk of neglected tropical disease infections and that the existence of such infections can itself lead to vitamin B12 deficiency (35). Given its association with an increased risk of adverse pregnancy outcomes and impaired psychomotor and cognitive development, and its role in the health of the elderly, vitamin B12’s public health impact is almost certainly underestimated (43). Similarly, vitamin E deficiency, with its role of counteracting oxidative stress, improving immune functions, and protecting against neurologic and cognitive deficits, is suggested to be underestimated, especially in LMICs, where it coexists with other oxidative stressors, such as malaria and HIV infection (38).

Thiamin deficiency is another public health concern in several LMICs that is considered to be not getting the attention it should (10, 11). Thiamin deficiency is known to cause or contribute to infant mortality and may also have lifelong neurodevelopmental consequences (11). Thiamin insufficiency (as assessed by low blood-thiamin statuses) is endemic among several Southeast Asian countries: Cambodia (70–100% of infants and 27–100% of reproductive-age women); Laos (13% of hospitalized infants); and Thailand (16–25% of children and 30% of elderly adults) (11). Thiamin deficiency accounts for up to 45% of deaths in children under 5 Cambodia, 34% of infant deaths in Laos, and 17% of infant deaths in Myanmar (10). Deficiencies also exist in Africa, Asia, and the Americas, but these instances have typically been isolated (11).

Nevertheless, the vitamins of most public health concern are vitamin A, folate, and—now receiving greater attention—vitamin B12 and vitamin D. Minerals and trace elements of global public health importance include iron, zinc, calcium, selenium, and maybe magnesium (2), and are not further discussed here. The inexactitude of the prevalence figures cited for vitamin deficiencies reflects the challenges to assessing the sizes of the problems (6). **Table 1** aims to give a summary of the more important vitamins to this review.

**TABLE 1:** Summary of prevalence, biomarkers, populations at greatest risk, health impact if not addressed, and public health strategies used in vitamins of public health significance, where known^1^

Vitamin	Prevalence (Virtually All More Common in LMICs)	Biomarkers (Reference)	Populations at Greatest Risk	Health Impact	Public Health Strategies
A (retinol, pre-vitamin A carotenes)	190 M (33.3%) preschool age; 19 M (15.3% pregnant women; <0.7 µmol/L)	Serum RBP, se/pl retinol, MRDR (#8 Tanumihardo et al. 2016)	Children <5, pregnant women	Decreased immune function, increased morbidity from infectious diseases (and blindness), and mortality	Supplementation, increased dietary diversity, fortification, and biofortification
Folate	N/A globally; United States: 24% pre-fortification, <1% post-fortification	RBC folate, (ancillary se folate, se Homocysteine; Bailey et al. 2015, 7)	Pregnant women, the fetus, adolescents, the elderly	Neural tube defects, anemia, chronic disease risk	Fortification, supplementation
B12	N/A globally; 10–30% in elderly (and higher in LMIC)	Se/pl B12, se holoTC, se MMA. pl tHcy (Allen et al. 2018, 9)	Elderly, vegetarian populations	Pernicious anemia, neurological involvement	Fortification, supplementation, dietary diversity, I/M injection
Thiamin (B1) and other B vitamins (riboflavin B2, niacin B3, pantothenic acid B5, pyridoxal B6, biotin B7, etc.)	N/A globally; probably widespread in poorer communities; often geographically constrained	Pl thiamine, pl ThMP, whole blood ThDP, ETK activity coefficient, etc. (but often clinical diagnosis; Johnson et al. 2019, 10; Whitfield et al. 2018, 11; McNulty et al. 2008, 42)	Infants, older infants, and children (neurological); adults (often alcohol induced); refugees	Increased mortality from infantile beriberi, ‘dry’ beriberi, Wernicke-Korskakoff Syndrome, pellagra (Niacin)	Fortification, supplementation (often over-the-counter and breakfast cereals, reduced processing of cereals)
D	Uncertain, but estimates vary widely. Said to be uncommon in affluent settings but NHANES estimates 40% of US population	Se vitamin D, (<50 µmol/L**;** Roth et al. 2018, 12)	Geographically defined, but in United States, African-Americans and Hispanics a have higher prevalence	Rickets, disturbed calcium homeostasis and bone metabolism, chronic disease risk	Fortification (especially oils and fats), supplementation, increased exposure to ultraviolet radiation

1 ETK, erythrocyte transketolase activity; holoTC, holotranscobalamin; I/M, intramuscular; LMICs, low- and middle-income countries; M, million; MMA, methylmalonic acid; MRDR, modified relative dose response; NHANES, National Health and Nutrition Examination Survey (USA); N/A, not available; pl, plasma; RBP, retinol-binding protein; se, serum; tHcy, total homocysteine; ThDP, thiamin diphosphate; TMP, whole blood thiamin monophosphate.

### Measuring the scope of the problem

A range of challenges gets in the way of accurate measurements of the prevalences of vitamin deficiencies. Prime amongst these are a lack of data because of infrequent or even no national surveys; geographic and situational factors (often leading to sub-national data standing in for nationally representative data); the use of imperfect biomarkers (e.g., in vitamin B12); a lack of agreement on the best measures, because of changing definitions and uncertain cutoffs (e.g., in vitamin D); and, in LMICs particularly, a lack of financial resources and laboratory and personnel capacities (2, 6).

Besides a not-infrequent lack of consensus on the best biomarkers to use and their cutoff values, even the biomarkers that are accepted can be influenced by inflammation and infection, the hydration status of the subject, the subject's age and gender, kidney function, the analytical method used (including time of drawing of blood), the capacity of the laboratory being used and its resources, and other factors, such as the season or times of annual dietary shortages (6). The size of the problem depends also on a recognition of the outcomes of deficiency, which may be subtle or manifest in ways not expected or looked for, such as when the vitamin is acting as a facilitator, as just 1 co-enzyme or co-factor to a particular biological/biochemical process. It has been recognized, for example, that vitamin B12 statuses differ by whether a woman is pregnant, lactating, elderly, or so on, compared with control women with equivalent nutrient intakes (44).

An example of this is vitamin A deficiency, where new evidence and interpretations of what actually constitutes a vitamin A deficiency have vastly expanded understandings of the prevalences of the deficiency, and so increased its public health importance (45). The deficiency had been recognized as a cause of night blindness and xerophthalmia at least since ancient Egypt, Greece, and Assyria, where it was treated with fish and animal liver and oil (46); was described clinically in the late 19^th^ century in Denmark, Japan, India, the United Kingdom, and the United States; and then was implicated in the mid-1980s in the increased risk of mortality in young children in LMICs with infectious diseases (45), although the mechanism was initially unclear. While the end stage of blindness is clearly an individual and family disaster, especially in poorer individuals and families, in global public health terms, there were many public health nutrition priorities competing for attention and resources, and vitamin A deficiency was deprioritized, given the relatively low prevalence of blindness (37, 45). Then, in the early 1980s, a program of providing high-dose vitamin A capsules to young Indonesian children was found to be saving children's lives, besides reducing ophthalmological signs such as night blindness and blindness from ulceration of the cornea through dryness (xerophthalmia) (45). While there was some initial resistance by the public health community to this finding, enough replications of the results in different settings allowed a consensus that something like a 24% reduction of young child deaths from infectious diseases in LMICs could be expected from vitamin A supplementation (47). This immediately, vastly increased the number of children diagnosed as vitamin A deficient, and so the deficiency came to be perceived as a far more significant public health concern. With an effective intervention now known and vastly increased numbers of children (and later, pregnant women in LMICs) defined as vitamin A deficient, the new emphasis on child survival by reducing the mortality rate in children under 5 years of age became an issue of importance to the UN Agencies, especially UNICEF, and vitamin A deficiency control and prevention programs were created by bilateral donors in Canada and the United States, who funded and provided expertise for national programs (mainly for supplementation) in many countries. A recent systematic review confirmed that vitamin A supplementation reduces the overall risk of death and deaths due to diarrhea by 12%, while reducing new episodes of diarrhea and measles (and blindness from vitamin A deficiency) (48).

With considerable success in many national programs, although the actual physiological mechanism remains unclear, there is now a suggestion by some that the impact is no longer as great as it was. This is, perhaps, because measles and diarrhoa are, globally, less of a problem due to immunization and health interventions such as oral rehydration supplements and zinc for diarrhea. The true impact is a continuing source of debate at present (49, 50) and has practical considerations in terms of what interventions countries should be using and how to know when it is appropriate to shift, for example, from supplementation to dietary interventions, including fortification, or to some mixture of approaches. In the meantime, coverage with vitamin A supplements, twice a year, is stagnant or even dropping (51), before anything has come to take their place on the same scale.

## Prevention and Control

As noted above, the widespread micronutrient deficiencies that are reported today in LMICs were once not uncommon in the poor urban and rural populations of Europe and the United States. Goiters, congenital iodine deficiency syndrome (then known as cretinism), anemia, rickets, pellagra, and xerophthalmia were common illnesses until the early 20^th^ century (36), and so there is public health experience in eliminating most of these deficiency diseases in the more affluent world (2). As seen in the vitamin A example, differing prevention and control activities vary according to the seriousness and extent of the public health problem, the populations affected, and changes in deficiency epidemiological profiles, varying outcomes because of the decline of associated diseases, improved methods of diagnosis, and the improved science of interventions and prevention. In the first 4 decades of the 20^th^ century, the scientific community aimed to separate and characterize the vitamins responsible for xerophthalmia, rickets, pellagra, scurvy, and beriberi. Later, in the 1940s, the dietary requirements that were not being fulfilled and led to these diseases received considerable attention (52). With the increasing realization that rarely do people on poor-quality diets have just 1 deficiency, the concept of addressing multiple micronutrient deficiencies, apart from the iodization of salt and vitamin A supplementation programs, is in increasing favor, as are using more than 1 approach and using both nutrition-specific and nutrition-sensitive interventions (2).

An increasingly pressing need is for sustainable programs and approaches, not least because bilateral and UN support is declining for many nutrition interventions, especially for single-vitamin deficiencies (53). Improved dietary diversity would be ideal, as supported by improved standards of living and, hence, diets and healthy environments. A complication here is that the poor diets of vulnerable children often are not providing vitamin A or other necessary vitamins. Diets in LMICs are described as being monotonous, of low dietary diversity, and of poor quality as regards vitamin content: for example, the vitamin A in animal-source foods is much more available physiologically but is also much more expensive (37, 54). Cheaper diets, consisting largely of dark green leafy vegetables and some fruits that supply beta-carotene (a vitamin A precursor), are a lot less effective in terms of vitamin A activity (maybe 12–21 times less available physiologically) (37). This problem is worsened by the concurrent infectious diseases often seen in the same populations, which reduce appetites (and so micronutrient content in the reduced diet), increase utilization of vitamins, and increase excretion of vitamins (37). In most cases, a mixture of the following four methods are most effective and generally should be employed concurrently, adapted to the local situation and populations (2, 53).

First, improved diets can be provided through appropriate agriculture and horticulture, nutrition education, home gardens, small livestock holdings, and food availability and accessibility, including storage and cooking methods (3). Second, where diets are inadequate for reasons of poverty, environmental stress, or for other reasons, supplementation programs have been successfully introduced to complement dietary sources. Third, in other countries, including affluent countries and LMICs, notably Guatemala, fortification (4, 36, 55) and, more recently, biofortification have been successful to a greater or lesser extent (56). Fourth, because of the association of deficiencies with diseases, poor living conditions, and other inequities, public health measures are necessary to complement any of the interventions; these measures may include reducing disease incidences, improving breast-feeding rates, better complementary feeding, increased immunization coverage, improved water and sanitation measures, improved antenatal and obstetric care, and so forth (53, 57). Ultimately, for sustainability of programs there is a need to reduce global and national inequities through political and socio-cultural change and, increasingly, action on climate change (58).

As noted, vitamin deficiency prevention and control programs often, and increasingly, include minerals and trace elements of public health concern, such as iodine, iron, and zinc. This is especially so now, with the increasing adoption of integrated approaches and nutrition-specific and nutrition-sensitive programming (59).

### Increasing dietary quality and diversity

Among the best known instances of dietary factors is the use of citrus fruits and other vitamin C–containing fruits and vegetables to prevent and treat the scourge of scurvy at sea: a deficiency disease that is said to have caused more deaths at sea than storms, shipwrecks, combat, and all other diseases combined (60). Often occurring with pellagra and beriberi, scurvy was the most feared risk at sea. More broadly, because more dietary improvement often builds on existing practices and culture, food-based approaches are more potentially sustainable (3). “Food-based approaches provide several micronutrients at once, they are viable, affordable and sustainable, they have social, cultural, economic and environmental benefits and they are local and not top-down,” as the FAO describes ways of improving diets (3).

The strategies to improve diets in resource-poor situations mean increasing the production and consumption of foods that are high in available micronutrients through agriculture and horticulture, small animal production or aquaculture, and, increasingly, but still limited, biofortification (54). Social and behavioral change communication (SBCC), when properly developed, increases cultural acceptability, helps a program be economically feasible, and contributes to sustainability (54, 61, 62). Food storage, processing, germination, cooking methods, and strategic food combinations can all be used to increase micronutrient retention and the bioavailability of plant foods, and have been found to have a relatively large, measurable impact (54, 63). Such strategies can be reinforced by including dietary enhancers of micronutrient absorption (such as vitamin C–containing foods) and by reducing absorption inhibitors (such as some forms of cassava). However, social change is often slow and requires appropriate formative research, and so, in the short-term, improving diets is often constrained by local limits of availability and accessibility. The limited research on the impact of dietary changes in such settings (including systematic reviews) has been inconclusive due to often weak methods of evaluation studies, although a major evaluation by Helen Keller International was conducted in 4 country programs and showed largely positive outcomes (62).

This showed improved household garden practices, food production, and consumption; increased dietary diversity and income; and reduced anemia among preschool children. The number of crop varieties consumed was significantly increased, including in the proportion of households consuming eggs and/or liver. Another evaluation showed increased empowerment among poor women with home gardens (64). Bangladeshi children who did not receive routine vitamin A supplementation were less likely to be night blind if the family had a home garden (64). In general, while a dietary approach (in its broadest definition) is most likely to ensure sustainable, if modest, impacts on micronutrient statuses, growth, and development, it needs to be combined with strategies that address the underlying causes of undernutrition, including reducing inequities and poverty alleviation, as well as promoting food security and income generation (61, 63, 64).

### Supplementation

The relative failure of large-scale protein-energy malnutrition interventions in the 1980s reflected the difficulties of enhancing dietary diversification and using social and political measures to make changes in nutrition outcomes at a large scale. These factors made vertical programs^1^ such as supplementation attractive, not least because of their cost-effectiveness (the Copenhagen Consensus concluded a benefit:cost ratio of 9.5:1) (27). To international, bilateral donors, such programs also appeared to offer potential solutions with a clear problem, a clear solution, and clear outcomes; with their funding and support, there were considerable successes in at-risk countries (47, 50).

While overall these programs showed successful impacts, some of the disadvantages of vertical programs became clearer with more experience, including the frequent dependence on external funding, for example, for vitamin A (Canadian Department of Foreign Affairs and Trade), iron/folic acid (UNICEF), and HIV programs (UNAIDS). Other challenges included logistics and supplies issues; the difficulty in reaching those most at risk due to their geographic and social isolation; and issues of program sustainability and policy transitions to other mechanisms. There was also a lack of proper national evaluations (48). Nevertheless, many millions of lives were, and continue to be, saved by the programs (48, 50, 65).

Recent reviews, noting the increased demands in pregnancy and the coexistence of deficiencies of multiple micronutrients, have found evidence that supports the use of multiple micronutrient supplements (rather than only the iron-folic acid supplements), as they have been shown to reduce the risk of low birth weights, preterm births, and infants being born small for their gestational age (15, 66). While this is currently not WHO policy, except under limited circumstances, the policy seems likely to change in the near future (67). Consequently, the use of multiple micronutrient supplements, as opposed to single vitamin supplements, seems the likely trend moving forwards (except perhaps for vitamin D (24), vitamin A, and iodine). Much of the more affluent world makes use of multivitamin supplementation for much less physiological reason or need (68), including in most pregnancies. Over half of the US population takes vitamin supplements, including 70% of those aged 60 and older: among these older adults, 29% take 4 or more supplements of any kind (24).

### Fortification

As noted above, vitamin deficiencies of vitamin A, vitamin D, niacin, and B vitamins were previously endemic in much of North America and Europe. During the 1930s, most of the chemical structures of the major vitamins were elucidated; most could be synthesized, thus enabling their addition to food (36, 52). Vitamin A was first added to margarine voluntarily in the United Kingdom in 1927, and it became a mandatory practice during the Second World War, largely to achieve nutritional equivalence of margarine to butter. After the introduction of vitamin A–fortified margarine in Denmark in 1917, the number of cases of xerophthalmia reported at Copenhagen Hospital fell by more than 90% and it had been eliminated by 1918 (69). The mandatory addition of vitamin D to milk that started in 1965 in Canada eliminated the widespread problem of childhood rickets (55). Voluntary enrichment of bread and other grain products with niacin was implemented in 1938 in the United States, with mandatory fortification following in 1940; as a result, pellagra had become almost nonexistent by 1950 (55). Iron, thiamin, niacin, and riboflavin were later all required to be added to wheat flour and other cereal products in North America and parts of Europe, to replace nutrients lost during the milling process and to reduce the risks of anemia, beriberi, pellagra, and riboflavin deficiency, respectively (36, 55, 70); this contributed to the virtual elimination of beriberi and pellagra in the more affluent world. Fortification has also been used in LMICs like Guatemala, where the early and historical fortification of sugar with vitamin A effectively controlled a previously serious problem in young children in a cost-effective, sustainable way (4, 55, 69).

Sustainability is more likely when the costs of fortification are passed onto the consumer and when changes in consumption habits are not required (71). Nevertheless, this does not mean that consumers do not need to be aware of their food supply being fortified, not least for continuing political support (4). However, in terms of effectiveness in LMICs, mandatory fortification generally requires centrally-processed food factories and the presence of strong compliance and regulation within the country. There are often limitations on reaching those most at risk of deficiency, because of geographic remoteness or a lack of accessibility to food due to poverty. There are also, in such settings, often challenges to assessing effectiveness. Aiming for sustainability, and to reach other populations in LMICs, the large-scale fortification of staple foods has been recommended by the WHO (71) and promoted by others, such as the Micronutrient Initiative (now Nutrition International), the Global Alliance for Improved Nutrition, and other civil society organizations (36, 55, 69). Various methodological recommendations and standards have been developed along with the promotion of fortified staple foods, especially by the WHO, the CDC, and the private sector (71). The effective reach of fortified of foods, especially staples, depends on both the distribution of the deficiency, the presence of a suitable food vehicle, the reach of the distribution of the fortified foods (4), and quality assurance and control programs (55). In the United States, mandatory, large-scale fortification of enriched cereal grain products with folic acid was authorized in 1996 and fully implemented in 1998. Within 5 years, the prevalence of neural tube defects had been dramatically reduced, to around 0.66/1000 pregnancies or less (69). The fortification of cereal grain products with folic acid became mandatory in several countries soon after, and has been consistently effective in reducing the prevalence of neural tube defects to an even lower incidence, of around 0.5 per 1000 total births, in countries where it has been implemented (69).

Where a deficiency is widespread in a population, large-scale fortification is most efficiently addressed if mandated by legislation (69, 71). Other, mainly affluent countries have had success with voluntary approaches and, in some cases, being even more targeted, increasingly by the adoption of commercial formulations of vitamin fortificants in food and drinks: often, these fortifications are more for a perception of improved wellbeing and for marginal health and functioning improvements, rather than for any real deficiency. Other methods of delivery have included so-called home/point-of-use fortification powders, such as Sprinkles (72); the more recent biofortification of sweet potatoes in Southeast Africa (73) and elsewhere; and, less successfully because of consumer and/or donor resistance, genetically modified plant fortifications, such as the development of “golden rice” (55). The widespread and often unrecognized endemicity of B vitamins and their interactions with other micronutrients (41) means that they are also routinely added to fortified, complementary feeding supplementary foods (thiamin, riboflavin, niacin, vitamin B6, and vitamin B12, along with folic acid, other micronutrients, and the macronutrients of energy, protein, carbohydrate, and fat) (70) when treating acute malnutrition in children. Commercially fortified young child–complementary foods are also extensively used in affluent countries and by LMICs’ elites, even when there is not a problem of vitamin deficiencies.

Large-scale fortification has used cereals (e.g., folic acid), wheat and maize flour, and, less successfully due to technical challenges, rice: the lack of success in rice is a pity, as it is consumed by more people globally. It has also been used in fats and oils, such as vegetable oils used for cooking, butter and margarine milk, and condiments such as salt (iodine), sugar (vitamin A), fish sauce, and soy sauce (36, 55), especially for the fat-soluble vitamins. Fortification with vitamins has been a challenging strategy, largely owing to sensory changes of the finished product and a poor stability of the fortificant when using certain vehicles (36, 74). There has, nonetheless, been notable success, and at least 23 countries now require fortification of edible oils (74). Modelling has also shown that the fortification of vegetable oil, rice, and sauces is an effective strategy to address micronutrient gaps and deficiencies in young children, including in LMICs such as Vietnam (75).

### Accompanying public health and nutrition measures

For both effectiveness and for sustained programs, addressing nutrition deficiencies requires looking beyond the specific vitamin or micronutrient. National programs need to take measures to include the whole diet and the environment in which the diet is being consumed, including by addressing infectious diseases; antenatal care; newborn, infant, and young child nutrition; water, sanitation, and hygiene improvements; social equity; and household food distribution (58). Addressing all these nutrition-sensitive influences on deficiencies will strengthen and sustain the nutrition-specific measures above (3). Women's (and young and adolescent girls’) education is among the most important factors (59).

## Political, Social, and Other Factors: Who is at Risk?

Women and young children, especially in LMICs, as well as adolescents, are particularly vulnerable to deficiencies, as are the increasing populations of the elderly. It is often said that “most people can get all the vitamins they need from a healthy diet,” but getting a healthy diet is often exactly the challenge, as diets in cash-strapped populations are often monotonous, less dense in micronutrients, and have few sources of vitamin- and micronutrient-rich animal-source foods. This paper addresses the populations at risk of deficiencies, rather than individuals with clinical deficiencies that might be found and dealt with in health facility and hospital conditions.

So, who is at risk? Those at most risk include sub-populations living in poverty and the food insecure: over 821 million people globally are considered to be food insecure (3). These populations include women; children and adolescents, as their growth patterns result in increased needs (2); those in emergencies and disasters (1); the elderly (76); and those living where there are high prevalences of clinical and disease conditions. Childhood infectious diseases can affect the vitamin intake through reduced appetites, poor physiologic absorption, and, often, such as in HIV and tropical enteropathy, increased utilization and excretion of vitamins. Even in affluent populations with high concentrations of chronic diseases and obesity, micronutrient deficiencies occur in resource-poor settings, including in urban settings (13, 77). A systematic review of all studies (1988–2008) reporting on micronutrient intakes in women living in resource-poor environments in the United Kingdom showed that, except for vitamin A (29%), vitamin C (34%), and niacin (34%), mean/median intakes of vitamins were below the estimated average requirements in over 50% of the studies (77). Pregnant women in LMICs are at especially high risk of micronutrient deficiencies and poor reproductive outcomes, as their poor diets lead to inadequate weight gain and multiple micronutrient deficiencies. Other negative factors include early marriages and adolescent pregnancies; poorly resourced health systems and inadequate obstetric care; multiple births and the close spacing of pregnancies; poor health; poor water and sanitation; and poverty, inequities, and some cultural practices, including when women have less access to education at all levels (18, 20).

### Economic impact

An early estimate by the World Bank suggested that 3–4% of gross domestic products may be lost to micronutrient deficiencies in LMICs (78). Estimates include acute impacts and longer-term developmental impairments that affect educational attainments and economic earning power. Just in LMICs alone, micronutrient deficiencies have been estimated to cause an annual gross domestic product loss of 2–5% (36), with direct costs estimated to be between US $20–30 billion every year (27). The potential of micronutrient interventions to reduce related diseases and their resultant economic burdens is well-recognized, but the information needed to inform cost-effectiveness needs improved tools that integrate information on program coverage, dietary intake distribution, biomarkers, and costs (6, 53).

## The Progress of Programs and Policies Addressing Vitamin Deficiencies

The greatest constraints in the changing world are the apparently intractable numbers of food-insecure persons in the world, with obvious consequences for vitamin deficiencies. Current estimates are of over 821 million people who are food (and nutritionally) insecure (3), with 108 million severely food insecure (up from the already alarming 80 million in 2015). At the same time, the overall global population is still increasing and there are growing constraints on land, water, and fisheries (58, 79). Climate change is already affecting the food supply, including the availability of micronutrients, especially iron and zinc, and, although probably less so, vitamins (79).

### Dietary adequacy and diversity

Over 800,000 households in Bangladesh have been reached with home gardens, which also empower poor women, and programs have also been established in parts of Southeast Asia and in West Africa (62). A recent review on sustainability concluded that is indeed possible to “improve the micronutrient density of the food supply by increasing fruit and vegetable and/or small-scale livestock through agricultural diversification” by promoting home gardening, especially when paired with nutrition and health SBCC (80).

However, the global challenges to food security remain: 1 person out of every 9 in the world is food insecure (3). The recent State of Food Security and Nutrition in the World report found that undernourishment and severe food insecurity appear to be increasing in almost all sub-regions of Africa, as well as in South America, whereas the undernourishment situation is stable in most regions of Asia. A more encouraging finding last year was that the rising trend in undernourishment had not yet been reflected in rates of child stunting; this was expected to continue to be the case in 2019 (3). FAO and its partners (International Fund for Agricultural Development, World Food Program, UNICEF, and WHO) have reiterated the call for a multisectoral approach to reduce undernutrition and the burden of stunting and wasting, in order to reduce childhood morbidity and mortality (3), reach global nutrition goals, and reach the second sustainable development goal.

### Supplementation

Again using vitamin A as an example, progress has been considerable via supplementation, using several different processes and varying staged events. Vitamin A deficiency considerably increases the mortality rate of measles in young children in LMICs, and the allied successes of measles vaccination programs and vitamin A supplementation programs has led to considerable decreases in the consequences of measles. The global progress of the vitamin A supplementation programs has been seen in the estimates of decreasing deaths, from 1.3 to 2.5 million preventable deaths in 1992 to approximately 650,000 deaths annually in 2003 (8); this trend that can be explained, in part, by reduced fatality rates of about 50% from measles and approximately 40% from severe diarrhea and dysentery, as a result of improved vitamin A statuses. This is a good example of different sectors working together on the prevention and control of vitamin deficiencies (49).

Supplementation programs had been steadily increasing their coverage, with 2 doses per year (47), although a recent report by UNICEF in 2018 showed some worrying backsliding internationally. The initial, positive trends were helped by linking vitamin A supplementation (VAS) to polio immunization on National Immunization Days (reaching coverage of 85%), and then was somewhat maintained by national vitamin A supplementation days or weeks (47). In terms of ongoing sustainability, there have been allied efforts to make supplementation days part of routine community and district care, and it was estimated that about 60% of eligible children were being reached. Since 2000, these global efforts to scale up VAS programs have yielded dramatic improvements in coverage from a decade before, contributing to a decline in child mortality (49, 81). But these efforts peaked at 78% (290 million children) in 2009 due to a loss of delivery platforms, weak health systems, and fragile states (50), leading to a shocking 6-year low. In 2016, 64% of children in need in priority countries were reached, but more than 140 million children were not, leaving them vulnerable to increased risks of disease and death (50).

### Fortification

In comparison, Guatemala, and subsequently other Central American countries, showed it was possible to virtually eliminate vitamin A deficiency by fortifying sugar with vitamin A (2, 4). Especially in Southeast Asia, commercial foods and oils are also increasingly being fortified (75). The effectiveness of large-scale fortification of staple foods to prevent micronutrient deficiencies in affluent countries has been demonstrated for over 80 years now, whereas the effectiveness in LMICs has been more recent. A recent review showed that measurable improvements in the micronutrient and health statuses of women and children in LMICs are now happening (82).

Currently, 86 countries mandate fortification with both iron and folic acid (except Australia, which does not mandate iron but fortifies with thiamin) and 4 mandate iron with no folic acid (Congo, Philippines, the United Kingdom, and Venezuela). Nearly a third (31.3%) of all the world's industrially-milled wheat flour is fortified, as is 64.6% of the world's industrially-milled maize flour and 0.8% of the world's rice (at this point only Papua New Guinea has mandated the fortification of rice) (83). There is now said to be increased effectiveness of receipt by target populations (as identified by the recently established Global Fortification Data Exchange). Even in countries like Afghanistan, which has a variety of constraints on extensive public health programs, there have been examples of voluntary fortification with vitamins (and other micronutrients), such that 71% of wheat flour is fortified with vitamins B9 (folic acid) and B12 (along with iron and zinc), and 33% of oil is voluntarily fortified with vitamins A and D (83).

## Discussion

### Emerging issues

Not all of the following issues are just emerging, but all require increased attention and resources. They include the identification of suitable, field-friendly, and innovative biomarkers; increased human and financial resources; and improved logistics at community, sub-national, and national levels (55). As the situations in countries change, concentrations of deficiencies change (including increasing in times of climate emergencies and human conflict), so that countries need to make decisions on modifications to programs, including determining when to transition to a different configuration of delivery mechanisms (53). A factor here is that, with increasing participation in the global market economy, there may be more sources of a particular vitamin than when the program was first started. In the case of vitamin A, periodic, high-dose supplementation was seen as a short-term solution until more sustainable solutions became available. In most countries with a public health problem, supplementation programs provide much of the protection, but now there is increased fortification of oil or other staples, such as sugar; improved diets as some countries become more affluent; and the availability of commercialized, common staples in breakfast cereals and snack foods. A recent examination of the potential for excess intakes has been undertaken by Tanumihardjo et al. (5) in Guatemala, South Africa, the United States, and Zambia, and there is indeed a potential for excessive intake in some groups.

Another concern is the micronutrient deficiencies occurring in obese individuals and in other noncommunicable diseases, where they may be overlooked, but more research is needed to contribute to public health approaches (25, 31). Already, much of the adult population in affluent countries is taking vitamin supplements, although there is skepticism as to whether their regular ingestion is worth it, both financially and nutritionally, or not (68). Climate change and its impact on micronutrient availability is an increasingly real, looming risk, but currently this appears to be more likely to affect the iron and zinc in cereals than to affect vitamins. Overall, increased resources are needed to gather more country data (including on the prevalence of deficiencies), including through applied research.

As in much of life, interest in scientific enquiry is somewhat at the call of fashion or enthusiasms. This can be driven by new discoveries, which open up other aspects of interest; by unrelated advances in different areas, such as genomics and the various other -omics; and even by increasing economic opportunities, as by donor priorities and by new initiatives and targets encouraged by the United Nations and other agencies. Again, vitamin A is something of an example: driven by a prominent university and supported by United States Agency for International Development, it allowed a real flowering of work in the 1980s and 1990s in epidemiology through the technical delivery of vitamin A in capsules and then through programmatic science, all supported technically by a special consultative group (International Vitamin A Consultative Group). This model was used for other vitamins and trace elements (such as zinc and, less successfully, iron deficiency in anemia). The role of folic acid in the fortification of cereals was helped by pressure from the Spina Bifida lobby group, as well as committed academics, especially at the CDC. As questions arose regarding vitamin A supplementation’s sustainability, more interest developed in the fortification of oils in LMICs. Going right back to earlier examples that led to the virtual elimination of pellagra and beriberi, army and navy recruits were fed polished rice from which the thiamin had been removed, which was driven to some degree by the economics of the health of workers in the deep South of the United States and by the defence needs of the Japanese (and others). There are many other examples (36, 55).

There are also commercial forces in desires for “optimal health,” however ill-defined, and again, some micronutrients move in and out of fashion. To give a not very scientific example, the University of Sydney Library was asked to look at changing publication rates over an arbitrary 20-year span. As can be seen in **Table 2**, the interest in vitamin A has somewhat tailed off, partly perhaps because of less funding being available, but also possibly because fortification is more a commercial enterprise than an academic one; the increase in interest in vitamin B12 might be hypothesized as being due to an increasingly large proportion of the population becoming elderly; vitamin C has shown little change, while interest in vitamin D has quadrupled, which is not surprising given the interest of the lay press; folate has seen a shift, perhaps because of the success of and questions about folic acid fortification and its longer-term impacts; and vitamin E, although not showing much change, could well reflect 2 peaks of interest, as 20 years ago there was considerable interest by some regarding its impact on some noncommunicable diseases (NCDs), and now there appears to be interest again, including in LMICs (38). These are broad observations, but a common factor is the presence of 1 or 2 charismatic figures advocating for and being involved in each wave. In commercially-fortified foods, public interest appears to have moved on somewhat from vitamins to the microbiome and probiotics.

**TABLE 2:** Change in number of articles over 20 years in a limited search^1^

Vitamin	1997	2017
A	723	583
B12	329	455
C	994	1109
D	673	2537
Folate	842	1381
E	1073	1381
Total articles	3906	6161

1 Data are from Medline searches by Rod Dyson, 29 May 2018.

In the BOND series, the expert panels were asked to look at outstanding research and development needs (6). The conclusions reached by the expert panels all noted the lack of functional biomarkers and the need to improve assay robustness, cost-effectiveness, and transportability to low-resource settings (7). One suggestion is for more information on the use of dried blood spots as an analytical tool. The folate expert group, while identifying other research gaps, called for simultaneous measurements of different but complementary biomarkers, giving examples such as DNA methylation, uracil in DNA, DNA or chromosomal breaks, and chromosomal breaks associated with folate deficiency or excess (7). Simultaneously, there is a need for confirmation of the possible negative outcomes of repeated consumption of high doses of commercial vitamins from various sources and, for folic acid, the consequences of long-term consumption through the fortification of cereals (9).

The research gaps and needs identified were purposefully directed at public health approaches. All 3 of the BOND expert groups identified the need to assist countries in knowing when to modify their programs and to determine which combinations of biomarkers can be used to characterize and then improve programs. For Vitamin A, there is still a need to better characterize the appropriate concentrations for serum retinol concentrations for infants, quite apart from the fact that serum retinol is known to be an imperfect measure of vitamin A in the body (8). Knowing that inflammation and infection affect serum retinol concentrations, how is this information best used to adjust, or not, when acute-phase proteins are measured (8)? Vitamin B12 has some somewhat similar problems in identifying the best biomarkers and identifying appropriate cutoff points (9). There is a need for an assessment of the functional significance of low B12 statuses in general, but especially in pregnancy, during lactation, during infancy, in young children, and especially in the elderly, where there are a myriad of questions about its function, how best to measure B12 concentrations and functions, and how to best treat apparent deficiencies (9). It is already known from research in Colombia that vitamin B12 is associated with grade repetition and school absenteeism (84): how big a problem is this, and how should it be addressed? A recent literature review of the current evidence called for more work on the role of vitamin B12 in neglected tropical diseases risks and severity, so as to inform interventions in at-risk populations beyond the accepted role with helminths (35).

Research questions around folate include its overlap with vitamin B12, such as in the elderly; the need for better biomarkers, especially those for individual pathways within the folate-mediated 1-carbon metabolic network; and validated biomarkers for the age-associated pathologies, such as neurodegeneration, cancers, and NCD (7). Attention to thiamin deficiencies in LMICs has recently increased, as there is increasing recognition that not only does it play a role in infant mortality, but that even a subclinical thiamin deficiency in childhood may have lifelong neurodevelopmental consequences (52). The harmonization of a robust study design to determine the genomic effects of depletion and repletion of folate has been called for, as was a gene expression network analysis to verify the mechanisms underlying observed genomic effects (7). All of these questions are but a few of the many proposed by the 3 expert groups (7–9).

Questions have also been asked regarding the public health importance of vitamin E in developing countries (38). Vitamin D research and knowledge, at present, epitomizes the need for better biomarkers, cutoff points, and understanding of the impacts of deficiencies. Complicated as they are by climatic and geographic and cultural factors, current estimates of the prevalence of vitamin D deficiency globally range from labeling it as infrequent to claiming it impacts virtually everybody, and the ranges of conditions thought to be related to vitamin D deficiency are equally wide (12). Prospective studies have found a positive association between low vitamin D concentrations and cardiovascular deaths, as well as all-cause mortality (85). A recent meta-analysis suggested that vitamin D supplementation at normal doses was associated with decreased all-cause mortality rates, but the conclusion was that, while vitamin D may play a part in multiple causes of death, “causality has not been determined” (86); as noted above, the recent VITAL study did not find that supplementation with vitamin D lowered the incidences of invasive cancer or cardiovascular events (32).

Given that the first vitamin was first discovered over a century ago, a surprising amount is still not known, and as new areas of research open up, much will continue to be explored. Applying this new knowledge, and existing knowledge, in public health programs, especially in LMICs, will be a major challenge, as will doing so in an equitable way. Already, the majority source of intake of some vitamins in children in Australia, some European countries, and North American countries is through commercially-fortified foods, such as breakfast cereals. A recent overview in Europe showed there is a surprisingly high prevalence of inadequate intakes of vitamins, but that voluntary fortification practices have been shown to improve the intake and status of key micronutrients in European Union population groups; however, these practices did not appear to contribute appreciably to risks of adverse effects from excess intakes (87).

### The elderly

Between 2015 and 2050, the proportion of the world's population over 60 years of age will nearly double, from 12% to 22%, and the pace of aging is increasing (76). The nutrition and health requirements of the elderly are as pressing in LMICs as in more affluent populations, as by 2050, 80% of older people will be living in LMICs (76, 88). A large proportion of older adults do not consume sufficient amounts of many nutrients, including micronutrients, from foods alone (88). There may also be issues with appetite, absorption, utilization, and excretion. Supplements in affluent populations compensate to some extent, with an estimated half of the elderly populations using them daily in many countries (89). Vitamin B12 and folate deficiencies are both of concern in elderly populations (9), as are other B vitamins, such as niacin and riboflavin, all of which may have increased needs. Vitamin D is frequently noted as an issue, for example, in frailty and mortality in populations (90). Vitamin K and vitamin E both may also be at risk. Not unrelated from these concerns of insufficiency, between 1990 and 2013, the number of deaths from NCDs has increased by 42%, with the largest increases in the proportion of global deaths occurring in the population aged 80 years and over (75, 88).

There is already a growing body of literature looking at the associations between NCDs and micronutrient deficiencies. Both have literature that suggest associations exist between micronutrient deficiencies and obesity in different populations, which may impair the immune system and increase the risk of comorbidities (25, 31, 91). There are also multiple pathways by which a micronutrient deficiency could impair appetite regulation and energy metabolism and could cause altered metabolism and excretion (31). These associations and possible mechanisms of the deficiencies' metabolic effects, such as their influence on leptin and insulin metabolism, need further studies to help clarify the roles of the different micronutrient deficiencies with respect to obesity and its comorbid conditions (91).

### Micronutrient goals

In the past, key international meetings with resultant targets (which have not always been realistic, but which could be considered aspirational) have been important contributing factors to the increased number of programs targeting vitamin and mineral deficiencies. The UN World Summit for Children in 1990 in New York established goals for vitamin A, iron, and iodine. This followed the “Hidden Hunger” Conference (organized by the Micronutrient Initiative in Ottawa), which effectively confirmed the place of the 3 micronutrients on the global agenda. Building on these was the 1992 FAO/WHO International Conference on Nutrition at FAO in Rome, which also had targets for all 3. The Millennium Development Goals in 2000 had no specific targets, but acted as a 10-year follow-up to the 1990 Summit. The 6 WHO targets endorsed by the World Health Assembly in 2013 include a target for a micronutrient-related condition (anemia), which is the only target in a direct sense. The second sustainable development goal that was agreed to, along with 17 others in New York in September 2015, was the only goal to address hunger and nutrition directly, but the targets are not specific for vitamins or, indeed, for micronutrients.

## Future Progress and Constraints

### Future progress

On the whole, there is likely to be continued progress, with an increased emphasis on women's health and nutrition, the elderly, and children and adolescents; indeed, an emphasis throughout life. An impressive study published in 2017 estimated the micronutrient density of the food supply, then the prevalence of inadequate intake of 14 micronutrients and the average prevalence of inadequate intakes, for all countries for the period between 1961 and 2011 (63). They concluded that, over this 50-year period, the estimated prevalence of inadequate intakes of micronutrients declined in all regions due to increased total production of food and/or micronutrient densities (63). Vitamin A, folate, riboflavin, and thiamin, along with 3 trace elements (calcium, iron, and zinc), were the vitamins with the lowest concentrations of adequacy, but strong differences were seen across countries and regions (63). Fortification will continue to scale up, including in commercially bought foods. There has been a move away from single-nutrient, vertical programs a,s the utility of integrated programs has become a higher priority and the multisectoral approach of combining nutrition-specific and nutrition-sensitive programs has gained attention and evidence (59).

### Future constraints

As noted, approximately 821 million people continue to suffer from chronic undernourishment. There are likely to be decreasing food supplies due to climate change and population pressures. Food prices and the demand for animal-source foods that are good sources of micronutrients will likely increase, with consequences for the environment. Focus and sustainability will be issues, especially as donors appear to be losing some attention to nutrition. And finally, resources in general, including for research, will have much competition. With the spread of fortification and commercial use of vitamins (and trace elements) in snack foods, cereals, and condiments, there is a reminder that, although programmatically challenging, targeting should be considered in LMIC populations (92) and the closer monitoring and evaluation of appropriate fortifications and overlapping interventions will be needed. However, limited resources, both human and physical, will be a continuing restraint in many countries (55, 69).

## Conclusions

There are some good nutrition and health successes, particularly in young child undernutrition, including with vitamins*.* As noted, the prevalences of inadequate intakes of 14 micronutrients have declined in all regions due to increased total production of food and/or micronutrient density (63), and food fortification is expanding globally, including in LMICs (69, 83, 93). Young child mortality and maternal mortality rates have substantially declined. Vitamin A capsule coverage vastly increased from the levels in 2000. Child education levels for girls are much improved, and will have both direct and delayed improvements for women's and children's health and nutritional status throughout life.

The encouraging trends in micronutrient adequacy have been especially strong in East and Southeast Asia, while weaker in South Asia and sub-Saharan Africa: the latter is the only region where micronutrient density has decreased (63). The food supply (as estimated by energy requirements) was predicted to be a continuing challenge in many countries; even where the energy concentrations are adequate, the micronutrient adequacy showed large variations. Fortification has reduced the estimated prevalence of inadequate micronutrient intakes in all low-income areas, except South Asia (63). Even in South and Southeast Asia, the growing importance of staple foods and condiments as ingredients in the food industry has been identified, with growing implications for fortification; the promotion of fortified foods (75, 93) is already leading to improved micronutrient status and health outcomes in some LMICs following large-scale fortification. Nevertheless, while increasing micronutrient density seems possible, overall dietary adequacy will continue to be a challenge for many countries and populations, especially given the likely further impact of climate change. Supplementation in public health programs will increasingly be limited to specific populations, such as micronutrients in antenatal care, as opposed to commercial, voluntary fortification through store-bought foods, where the ability to access such fortified foods may be a constraint. At the same time, while it is known that dietary quality can be improved through fortification, biofortification, and agricultural efforts, as well as complemented by supplementation, it is necessary to increase the nutrition knowledge of the target populations, SBCC, and supportive policy, as well as reduce poverty and increase education, especially in women.

## Footnotes

1 Those programs and policies, usually created through external funding, that addressed a single health problem from global identification to interventions by addressing just the problem, rather than taking a wider systems approach.

## Notes

This article was presented in full at the 5th International Vitamin Conference in Sydney, Australia, August 8-10, 2018.

The authors reported no funding received for this study.

Author disclosures: ID-H, no conflicts of interest.

Abbreviations used: BOND, Biomarkers of Nutrition for Development; NCDs, noncommunicable diseases; LMIC, low- and middle-income countries; SBCC, social and behavioral change and communication; VAS, vitamin A supplementation.

## References

[1] Bailey RL, West KP Jr, Black RE The epidemiology of global micronutrient deficiencies. Ann Nutr Metab. 2015;66(Suppl 2):22–33. 10.1159/000371618 26045325

[2] Darnton-Hill I. Chapter 2, Prevalence, causes and consequences of micronutrient deficiencies. In Mannar V Hurrell R, editors. Food fortification in a globalized world. London, UK: Academic Press/Elsevier; 2017:13–28.

[3] FAO, IFAD, UNICEF, WFP, WHO The state of food security and nutrition in the world [Internet]. Rome, Italy: Food and Agricultural Organization of the UN; 2018 Available from:www.fao.org/3/i9553en/i9553en.pdf.

[4] Dwyer JT, Wiemer KL, Dary O, Keen CL, King JC, Miller KB, Philbert MA, Tarasuk V, Taylor CL, Gaine PC et al. Fortification and health: Challenges and opportunities. Adv Nutr. 2015;6(1):124–31. 25593151 10.3945/an.114.007443 PMC4288271

[5] Tanumihardjo SA, Kaliwile C, Boy E, Dhansay MA, van Stuijvenberg M Overlapping vitamin A interventions in the United States, Guatemala, Zambia, and South Africa: Case studies: Excessive intakes due to overlapping programs. Ann NY Acad Sci. 2018;83:1–56. doi: 10.1016/bs.afnr.2017.11.001 (Epub 2018 Feb 2). 10.1111/nyas.13965 PMC7999526 30265402

[6] Raiten DJ, Namasté S, Brabin B, Combs G Jr, L'Abbé MR, Wasantwisut E, Darnton-Hill I Executive summary: Biomarkers of nutrition for development (BOND): Building a consensus. Am J Clin Nutr. 2011;94(2):633S–50S. 21733880 10.3945/ajcn.110.008227 PMC3142731

[7] Bailey LB, Stover PJ, McNulty H, Fenech MF, Gregory JF III, Mills JL, Pfeiffer CM, Fazili Z, Zhang M, Ueland PM et al. Biomarkers of nutrition for development— Folate review. Supplement: Biomarkers of nutrition for development (BOND) expert panel reviews, Part 2. J Nutr. 2015;145(7):1636S–80S. 26451605 10.3945/jn.114.206599 PMC4478945

[8] Tanumihardjo SA, Russell RM, Stephensen CB, Gannon BM, Craft NE, Haskell MJ, Lietz G, Schulze K, Raiten DJ Biomarkers of nutrition for development (BOND) -Vitamin A review. J Nutr. 2016;146(9):1816S–48. 27511929 10.3945/jn.115.229708 PMC4997277

[9] Allen LH, Miller JW, de Groot L, Rosenberg IH, Smith AD, Refsum H, Raiten DJ Biomarkers of nutrition for development (BOND): Vitamin B12. J Nutr. 2018;148(Suppl 4):1995S–2027S. 30500928 10.1093/jn/nxy201 PMC6297555

[10] Johnson CR, Fischer TD, Thacher TD, Topzian MD, Bourassa M, Combs GF. Thiamin deficiency in low- and middle-income countries: Disorders, prevalences, previous interventions and current recommendations. Nutr Health. 2019;25(2):127–51. 30798767 10.1177/0260106019830847

[11] Whitfield KC, Bourassa MW, Adamolekun B, Bergeron G, Bettendorff L, Brown KH, Cox L, Fattal‐Valevski A, Fischer PR, Frank EL et al. Thiamine deficiency disorders: Diagnosis, prevalence, and a roadmap for global control programs. Ann NY Acad Sci. 2018;1430:3–43. 30151974 10.1111/nyas.13919 PMC6392124

[12] Roth DE, Steven A, Abrams SA, Aloia J, Bergeron G, Bourassa MW, Brown KH, Calvo MS, Cashman KD, Combs G et al. Global prevalence and disease burden of vitamin D deficiency: A roadmap for action in low‐ and middle‐income countries. Ann NY Acad Sci. 2018;1430:44–79. 30225965 10.1111/nyas.13968 PMC7309365

[13] Pijls L, Ashwell M, Lambert J EURRECA - A network of excellence to align European micronutrient recommendations. Food Chemistry. 2009;113:745–53.

[14] Shiel WC Jr. Vitamins [Internet]. MedicineNet. Available from: https://www.medicinenet.com.

[15] Lonsdale D. Thiamin. Adv Food Nutr Res. 2018;83:1–56. 29477220 10.1016/bs.afnr.2017.11.001

[16] Gernand AD, Schulze KJ, Stewart CP, West KP Jr, Christian P Micronutrient deficiencies in pregnancy worldwide: Health effects and prevention. Nat Rev Endoc. 2016;12:274–89. 10.1038/nrendo.2016.37 PMC4927329 27032981

[17] Shi H, Enriquez A, Rapadas M, Martin EM, Wang R, Moreau J, Lim CK, Szot JO, Ip E, Hughes JN et al. NAD deficiency, congenital malformations, and niacin supplementation. N Engl J Med. 2017;377:544–52. 28792876 10.1056/NEJMoa1616361

[18] Allen LH. Multiple micronutrients in pregnancy and lactation: An overview. Am J Clin Nutr. 2005;81(5):1206S–12S. 15883453 10.1093/ajcn/81.5.1206

[19] Keats EC, Haider BA, Tam E, Bhutta ZA Multiple-micronutrient supplementation for women during pregnancy. Cochrane Database Syst Rev. 2019;3:CD004905. 30873598 10.1002/14651858.CD004905.pub6 PMC6418471

[20] Darnton-Hill I, Mkparu U. Micronutrients in pregnancy in low- and middle-income countries. Nutrients. 2015;7:1744–68. 25763532 10.3390/nu7031744 PMC4377879

[21] Webb P, Nishida C, Darnton-Hill I Age and gender as factors in the distribution of global micronutrient deficiencies. Nutr Rev. 2007;65:233–45. 17566549 10.1111/j.1753-4887.2007.tb00300.x

[22] Darnton-Hill I, Webb P, Harvey PWJ, Hunt JM, Dalmiya N, Chopra M, Ball MJ, Bloem MW, de Benoist B Micronutrient deficiencies and gender: Social and economic costs. Am J Clin Nutr. 2005;819(Suppl 1):1198S–205. 10.1093/ajcn/81.5.1198 15883452

[23] Wieser S, Plessow R, Eichler K, Malek O, Capanzana MV, Agdeppa I, Bruegger U Burden of micronutrient deficiencies by socio-economic strata in children aged 6 months to 5 years in the Philippines. BMC Publ Health. 2013;13:1167:1–15. 10.1186/1471-2458-13-1167 PMC3867423 24330481

[24] Gahche JJ, Bailey RL, Potischman N, Dwyer JT Dietary supplement use was very high among older adults in the United States in 2011–2014. J Nutr. 2017;147:1968–76. 28855421 10.3945/jn.117.255984 PMC5610553

[25] Li Y, Wang C, Zhu K, Feng RN, Sun CH Effects of multivitamin and mineral supplementation on adiposity, energy expenditure and lipid profiles in obese Chinese women. Internat J Obesity. 2010;34:1070–7. 10.1038/ijo.2010.14 20142823

[26] Darnton-Hill I. Global burden and significance of multiple micronutrient deficiencies in pregnancy. In Bhutta ZA Hurrell RF, Rosenfeld IF, editors. Meeting micronutrient requirements for health and development. Vol 70 Nestlé Nutr Workshop Ser. Basel, New York: Karger; 2012:49–60. 10.1159/000337421 25825295

[27] Horton S, Alderman H, Rivera J Copenhagen consensus 2008. Malnutrition and hunger. Copenhagen, Denmark: Copenhagen Consensus Center; 2008.

[28] Bloem MW, de Pee S, Darnton-Hill I Chapter 27, Micronutrient deficiencies and maternal thinness. First chain in the sequence of nutritional and health events in economic crises. In: Bendich A, Deckelbaum RJ, editors. Preventive nutrition. The comprehensive guide for health professionals. 3rd edition. Totowa, NJ: Humana Press, 2005;689–710.

[29] Darnton-Hill I, Cogill B. Maternal and young child nutrition adversely affected by external shocks such as increasing global food prices. J Nutr. 2010;140:162S–9. 19939995 10.3945/jn.109.111682

[30] Hossain-nezhad A, Holick MF. Vitamin D for health: A global perspective. Mayo Clin Proc. 2013;88:720–55. 23790560 10.1016/j.mayocp.2013.05.011 PMC3761874

[31] Astrup A, Bügel S. Micronutrient deficiency in the aetiology of obesity. Internat J Obesity. 2010;34:947–8. 10.1038/ijo.2010.81 20543852

[32] Manson JE, Cook NR, Lee I-M, Christen W, Bassuk SS, Mora S, Gibson H, Gordon D, Copeland T, Copeland T, D'Agostino D et al.; for the Vitamin D and Lifestyle (VITAL) Research Group. Vitamin D supplements and prevention of cancer and cardiovascular disease. N Engl J Med. 2019;380(1):33–44. 30415629 10.1056/NEJMoa1809944 PMC6425757

[33] Kanatani KT, Nakayama T, Adachi Y, Hamazaki K, Onishi K, Konishi Y, Kawanishi Y, Go T, Sato K, Kurozawa Y et al. High frequency of vitamin D deficiency in current pregnant Japanese women associated with UV avoidance and hypo-vitamin D diet. PLOS One. 2019;14(3):e0213264. 30830935 10.1371/journal.pone.0213264 PMC6398852

[34] Darnton-Hill I, Ahmed FA, Samman S Chapter 30, The impact of micronutrients on inflammation and health in low- and middle-income countries. In Bendich A, Deckelbaum RJ, editors. Preventive nutrition. 5thed Totoya, NJ: Humana Press; 2015:597–644.

[35] Layden AJ, Täse K, Finkelstein JL Neglected tropical diseases and vitamin B12: A review of the current evidence. Trans R Soc Trop Hyg. 2018;112:423–35. 10.1093/trstmh/try078 PMC6457089 30165408

[36] Mannar V, Hurrell R, editors. Food fortification in a globalized world. London, UK: Academic Press/Elsevier; 2017.

[37] Palmer A, Darnton-Hill I, West KW Jr Vitamin A deficiency. In de Pee S Taren D Bloem MW editors. Nutrition and health in a developing world. 3rd edition Totoya, NJ: Humana Press; 2017:181–234.

[38] Dror DK, Allen LH. Vitamin E in developing countries. Food Nutr Bull. 2011;32:1124–43. 10.1177/156482651103200206 22164974

[39] Petry N, Olofin I, Hurrell RF, Boy E, Wirth JP, Moursi M, Donahue AM, Rohner F The proportion of anemia associated with iron deficiency in low, medium, and high human development index countries: A systematic analysis of national surveys. Nutrients. 2016;8(11):E693. 27827838 10.3390/nu8110693 PMC5133080

[40] Watson JT, El Bushra H, Lebo EJ, Bwire G, Kiyengo J, Emukule G, Omballa V, Tole J, Zuberi M, Breiman RF et al. Outbreak of beriberi among African Union troops in Mogadishu, Somalia. PLOS One. 2011;6(12):e28345. 22205947 10.1371/journal.pone.0028345 PMC3244391

[41] Powers HJ. Riboflavin (vitamin B-2) and health. Am J Clin Nutr. 2003;77:1352–60. 12791609 10.1093/ajcn/77.6.1352

[42] McNulty H, Scott JM. Intake and status of folate and related B vitamins: Considerations and challenges in achieving optimal status. Br J Nutr. 2008;99(Suppl 3):S48–54. 18598588 10.1017/S0007114508006855

[43] Allen LH. How common is vitamin B12 deficiency? Am J Clin Nutr. 2009;89:693S–6. 19116323 10.3945/ajcn.2008.26947A

[44] Bae S, West AA, Yan J, Jiang X, Perry CA, Malysheva O, Stabler SP, Allen RH, Caudill MA Vitamin B-12 status differs among pregnant, lactating, and control women with equivalent nutrient intakes. J Nutr. 2015;145(7):1507–14. 25995278 10.3945/jn.115.210757

[45] Sommer A, West KP Jr Vitamin A deficiency: health, survival, and vision. New York, NY: Oxford University Press; 1996.

[46] Wolf G. A history of vitamin A and retinoids. FASEB J. 1996;10:1102–7. 8801174 10.1096/fasebj.10.9.8801174

[47] Beaton GH, Martorell R, Aronson KJ, Edmonston B, McCabe G, Ross AC, Harvey B Effectiveness of vitamin A supplementation in the control of young child morbidity and mortality in developing countries [ACC/SCN State of the Art Ser, Nutrition Policy Discussion Paper No. 13]. Geneva, Switzerland: ACC/SCN; 1993.

[48] Imdad A, Mayo-Wilson E, Herzer K, Bhutta ZA Vitamin A supplementation for preventing morbidity and mortality in children from six months to five years of age. Cochrane Database of Systematic Reviews. 2010;(12):CD008524 doi: 10.1002/14651858.CD008524.pub3. 28282701 10.1002/14651858.CD008524.pub3 PMC6464706

[49] Mason J, Greiner T, Shrimpton R, Sanders D, Yukich J Vitamin A policies need rethinking. Int J Epidemiol. 2015;44:283–92. 25306559 10.1093/ije/dyu194

[50] Raiten DR, Darnton-Hill I, Tanumihardjo SA, Suchdev PS, Udomkesmalee E, Martinez C, Mazariegos D, Mofu M, Kraemer K, Martinez H Integration to implementation (I-to-I) and the micronutrient forum II: Addressing the safety and effectiveness of Vitamin A supplementation. Adv Nutr. 2019 In press. 10.1093/advances/nmz100 PMC7442412 31566677

[51] UNICEF Coverage at a crossroads: New directions for vitamin A supplementation programmes. New York, NY: UNICEF; 2018.

[52] Semba RD Thought regarding multiple micronutrient nutrition. J Nutr. 2012;142:143S–56. 22157539 10.3945/jn.110.137745

[53] Bruins MJ, Kupka R, Zimmermann MB, Lietz G, Engle-Stone R, Kraemer K Maximizing the benefits and minimizing the risks of intervention programs to address micronutrient malnutrition: symposium report. Maternal Child Nutr. 2016;12:940–8. 10.1111/mcn.12334 PMC5095875 27501994

[54] Gibson RS. Enhancing the performance of food-based strategies to improve micronutrient status and associated health outcomes in young children from poor-resource households in low-income countries: Challenges and solutions. FAO/CABI 2014. In Thompson B Amoroso L, editors. Improving diets and nutrition: Food-based approaches. Rome, Italy: The Food and Agricultural Organization of the United Nations; 2014:19–31.

[55] Micronutrient Forum (Darnton-Hill I, Neufeld L, Vossenaar M, Osendarp S, Martinez H). Large-scale food fortification: An overview of trends and challenges in low- and middle-income countries in 2017 [Internet; ISBN: 978-0-9959892-2-1]. Ottawa, Canada: Micronutrient Forum; 2017 Available from:http://micronutrientforum.org/content/user_files/2017/10/2017-09MNForum-LargeScaleFortification-FinalReport.pdf.

[56] Bouis HE, Saltzman A Improving nutrition through biofortification: A review of evidence from HarvestPlus, 2003 through 2016. Glob Food Security. 2017;12:49–58, E12. 10.1016/j.gfs.2017.01.009 PMC5439484 28580239

[57] Humphrey JH, Mbuya MNN, Ntozini R, Moulton LH, Stoltzfus RJ, Tavengwa NV, Mutasa K, Majo F, Mutasa B, Mangwadu G et al. Independent and combined effects of improved water, sanitation, and hygiene, and improved complementary feeding, on child stunting and anemia in rural Zimbabwe: A cluster-randomised trial. Lancet Glob Health. 2019;3(2):77–90. 10.1016/S2352-4642(18)30340-7 PMC6472652 30573417

[58] Raiten DJ, Combs GF Nutritional ecology: Understanding the intersection of climate/environmental change, food systems and health. CAB International 2019 In Fan S Yosef S Pandya-Lorch R, editors. Agriculture for improved nutrition: Seizing the momentum; 2019:68–80.

[59] Ruel MT, Alderman H Nutrition-sensitive interventions and programmes: How can they help to accelerate progress in improving maternal and child nutrition? Lancet. 2013;382(9891):536–51. 23746780 10.1016/S0140-6736(13)60843-0

[60] Vilter RW. A book review of ‘The History of Scurvy and Vitamin C by Kenneth J. Carpenter, ed. Cambridge: Cambridge University Press, 1986:288pp’ J Nutr. 1987;117:599.

[61] Thompson B Amoroso L , editors Improving diets and nutrition: Food-based approaches. Rome, Italy: The Food and Agricultural Organization of the United Nations, FAO/CABI; 2014.

[62] Talukder A, Akoto K, Osei AK, Haselow NJ, Kroeun H, Uddin A, Quinn V. Contribution of homestead food production to improved household food security and nutrition status – Lessons learned from Bangladesh, Cambodia, Nepal and the Philippines. In Thompson B Amoroso L, editors. Improving diets and nutrition: Food-based approaches. Rome, Italy: The Food and Agricultural Organization of the United Nations, FAO/CABI; 2014:58–73.

[63] Beal T, Massiot E, Arsenault JE, Smith MR, Hijmans RJ Global trends in dietary micronutrient supplies and estimated prevalence of inadequate intakes. PLOS One. 2017;12(4):e0175554. 28399168 10.1371/journal.pone.0175554 PMC5388500

[64] Darnton-Hill I. Chapter 7, The under-estimated impact of food-based interventions. In Thompson B Amorosos L, editors. Improving diets and nutrition: Food-based approaches. Rome, Italy: The Food and Agricultural Organization of the United Nations, FAO/CABI; 2014:74–88.

[65] Bhutta ZA, Das JK, Rizvi A, Gaffey MF, Walker N, Horton S, Webb P, Lartey A, Black RE; Lancet Nutrition Interventions Review Group; The Maternal and Child Nutrition Study Group. Evidence-based interventions for improvement of maternal and child nutrition: What can be done and at what cost? Lancet. 2013;382:452–77. 23746776 10.1016/S0140-6736(13)60996-4

[66] Smith ER, Shankar AH, Wu LS-F, Aboud S, Adu-Afarwuah S, Ali H, Agustina R, Arifeen S, Ashorn P, Bhutta ZA et al. Modifiers of the effect of maternal multiple micronutrient supplementation on stillbirth, birth outcomes, and infant mortality: A meta-analysis of individual patient data from 17 randomised trials in low-income and middle-income countries. Lancet Glob Health. 2017;5(11):PE1090–E1100. 10.1016/S2214-109X(17)30371-6 29025632

[67] Sudfield CR, Smith ER. New evidence should inform WHO guidelines on multiple micronutrient supplementation in pregnancy. J Nutr. 2019;149:359–61. 30773589 10.1093/jn/nxy279 PMC6398379

[68] Guallar E, Stranges S, Mulrow C, Appel LJ, Miller ER III Enough is enough: Stop wasting money on vitamin and mineral supplements. Ann Inter Med. 2013;159:850–1. 10.7326/0003-4819-159-12-201312170-00011 24490268

[69] Osendarp SJM, Martinez H, Garrett GS, Neufeld LM, De-Regil LM, Vossenaar M, Flores-Ayala R, Darnton-Hill I Large-scale food fortification in low- and middle-income countries: A review of programs, trends, challenges and evidence-gaps. Food Nutr Bull. 2018;39:315–31. 29793357 10.1177/0379572118774229 PMC7473077

[70] Eggersdorfer M Kraemer K Cordaro JB Fanzo J Gibney M, Kennedy E, editors. Good nutrition: Perspectives for the 21^st^ century. Basel: Karger; 2016.

[71] Allen L, de Benoist B, Dary O, Hurrell R Guidelines on food fortification with micronutrients. Geneva, Switzerland: World Health Organization; 2006.

[72] de Pee S, Moench-Pfanner R, Martini E, Zlotkin S, Darnton-Hill I, Bloem M Home-fortification in emergency response and transition programming: Experiences in Aceh and Nias, Indonesia. Food Nutr Bull. 2007;28:189–97. 24683678 10.1177/156482650702800208

[73] Low J, Mwanga ROM, Andrade M, Carey E, Ball A-M Tackling vitamin A deficiency with biofortified sweetpotato in sub-Saharan Africa. Global Food Security. 2017;14:23–30. 28989861 10.1016/j.gfs.2017.01.004 PMC5614018

[74] González de Mejia E, Aguilera-Gutiérrez Y, Martin-Cabrejas MA, Mejia LA Industrial processing of condiments and seasonings and its implications for micronutrient fortification. Ann NY Acad Sci. 2015;1357:8–28. 26312771 10.1111/nyas.12869

[75] Laillou A, Mai LB, Hop LT, Khan NC, Panagides D, Wieringa F, Berger J, Moench-Pfanner R An assessment of the impact of fortification of staples and condiments on micronutrient intake in young Vietnamese children. Nutrients. 2012;4:1151–70. 23112906 10.3390/nu4091151 PMC3475228

[76] WHO. Ageing and health. Geneva, Switzerland: World Health Organization; 2018 Available from: htpps://www.who.int.

[77] Torheim LE, Ferguson EL, Penrose K, Arimond M Women in resource-poor settings are at risk of inadequate intakes of multiple micronutrients. J Nutr. 2010;140(11):S2051–S8. 10.3945/jn.110.123463 20881075

[78] The World Bank Enriching lives: Overcoming vitamin and mineral malnutrition in developing countries [Development in Practice Series]. Washington, DC: World Bank; 1994.

[79] Myers SS, Smith MR, Guth S, Golden CD, Vaitla B, Mueller ND, Dangour AD, Huybers P Climate change and global food systems: Potential impacts on food security and undernutrition. Ann Rev Public Health. 2017;38:259–77. 28125383 10.1146/annurev-publhealth-031816-044356

[80] Frison EA, Cherfas J, Hodgkin T Agricultural biodiversity is essential for a sustainable improvement in food and nutrition security. Sustainability. 2011;3:238–53.

[81] Dalmiya N, Palmer A, Darnton-Hill I Sustaining vitamin A supplementation requires a new vision. Lancet. 2006;368;1052–4. 16997651 10.1016/S0140-6736(06)69336-7

[82] Keats EC, Neufeld LM, Garrett GS, Mbuya MNN, Bhutta ZA Improved micronutrient status and health outcomes in low- and middle-income countries following large-scale fortification: Evidence from a systematic review and meta-analysis. Am J Clin Nutr. 2019;109 (6):1696–1708. 30997493 10.1093/ajcn/nqz023 PMC6537942

[83] Food Fortification Initiative Global progress of industrially milled cereal grains. Available from:https://ffinetwork.org.

[84] Duong M-C, Mora-Plazas M, Marín C, Villamor E Vitamin B-12 deficiency in children is associated with grade repetition and school absenteeism, independent of folate, iron, zinc, or vitamin A status biomarkers. J Nutr. 2015;145;1541–8. 25972530 10.3945/jn.115.211391

[85] Dobnig H, Pilz S, Scharnagl H, Renner W, Seelhorst U, Wellnitz B, Kinkeldei J, Boehm BO, Weihrauch G, Maerz W Independent association of low serum 25-hydroxyvitamin D and 1,25-dihydroxyvitamin D levels with all-cause and cardiovascular mortality. Arch Intern Med. 2008;168:1340–9. 18574092 10.1001/archinte.168.12.1340

[86] Kulie T, Groff A, Redmer J, Hounshell J, Schrager S Vitamin D: an evidence-based review. J Amer Board Family Med. 2009;22:698–706. 10.3122/jabfm.2009.06.090037 19897699

[87] Hennessy Á, Walton J, Flynn A The impact of voluntary food fortification on micronutrient intakes and status in European countries: A review. Proc Nutr Soc. 2013;72(4):433–40. 24020749 10.1017/S002966511300339X

[88] United States Census Bureau (He W Goodkind D Kowal P). An aging world: 2015 international population reports [Issued March 2016 P95/16-1]. https://www.census.gov/library/publications/2016/demo/P95-16-1.html.

[89] Sebastian RS, Cleveland LE, Goldman JD, Moshfegh AJ Older adults who use vitamin/mineral supplements differ from nonusers in nutrient intake adequacy and dietary attitudes. J Am Diet Assoc. 2007;107:1322–32. 17659898 10.1016/j.jada.2007.05.010

[90] Smit E, Crespo CJ, Michael Y, Ramirez-Marrero FA, Brodowicz GR, Bartlett S, Andersen RE The effect of vitamin D and frailty on mortality among non-institutionalized US older adults. Eur J Clin Nutr. 2012;66:1024–8. 22692022 10.1038/ejcn.2012.67

[91] García OP, Long KZ, Rosado JL Impact of micronutrient deficiencies on obesity. Nutr Rev. 2009;67(10):559–72. 19785688 10.1111/j.1753-4887.2009.00228.x

[92] Baye K. Maximising benefits and minimising adverse effects of micronutrient interventions in low- and middle-income countries. Proc Nutr Soc. 2019;1–7. doi:10.1017/S0029665119000557 (Epub ahead of print). 10.1017/S0029665119000557 30853033

[93] Sporer R, Larson M, Maurin C, Laillou A, Capanzana M, Garrett GS The growing importance of staple foods and condiments used as ingredients in the food industry and implications for large-scale food fortification programs in Southeast Asia. Food Nutr Bull. 2013;34(Suppl 2):S50–61. 24049996 10.1177/15648265130342S107

